# Baseline Characteristics and Associated Factors of Mortality in COVID-19 Patients; an Analysis of 16000 Cases in Tehran, Iran

**Published:** 2020-09-06

**Authors:** Alireza Zali, Saeid Gholamzadeh, Gohar Mohammadi, Mehdi Azizmohammad Looha, Forouzan Akrami, Elaheh Zarean, Reza Vafaee, Ali Maher, Mahmood Khodadoost

**Affiliations:** 1 Functional Neurosurgery Research Center, Shohada Tajrish Neurosurgical Center of Excellence, Shahid Beheshti University of Medical Sciences, Tehran, Iran.; 2 Vice Chancellor in Administration and Resources Development affairs, Shahid Beheshti University of Medical Sciences, Tehran Iran.; 3 Legal medicine Research Center, Legal medicine organization, Tehran, Iran.; 4 Cancer Research Center, Shahid Beheshti University of Medical Sciences, Tehran, Iran**.**; 5 Medical Ethics and Law Research Center, Shahid Beheshti University of Medical Sciences, Tehran, Iran.; 6 Modeling in Health Research Center, Shahrekord University of Medical Sciences, Shahrekord, Iran.; 7 Proteomics Research Center, Student Research Committee, Shahid Beheshti University of Medical Sciences, Tehran, Iran.; 8 Department of Health Policy, Economics and Management, School of Management and Medical Education, Shahid Beheshti University of Medical Sciences, Tehran, Iran.; 9 School of Traditional Medicine, Traditional Medicine & Materia Medica Research Center Shahid Beheshti University of Medical Sciences, Tehran, Iran.

**Keywords:** COVID-19, Inpatients, Survival, Mortality, Comorbidity, Noncommunicable Diseases, Iran

## Abstract

**Introduction:**

**Introduction:**

Given the importance of evidence-based decision-making, this study aimed to evaluate epidemiological and clinical characteristics as well as associate factors of mortality among admitted COVID-19 cases.

**Methods:**

This multicenter, cross-sectional study was conducted on confirmed and suspected COVID-19 cases who were hospitalized in 19 public hospitals affiliated to Shahid Beheshti University of Medical Sciences (SBMU), Tehran, Iran, between February 19 and May 12, 2020. Epidemiological and clinical characteristics of the infected cases were compared between the deceased and survivors after discharge. Case fatality rates (CFRs) were calculated across all study variables. Single and multiple logistic regressions were used to explore the risk factors associated with COIVD-19 mortality.

**Results:**

Out of the 16035 cases that referred to the hospitals affiliated to SBMU, 16016 patients (99.93% of Confirmed and 99.83% of suspected cases) were hospitalized. 1612 patients died with median hospitalization days of 5 (interquartile range (IQR): 2-9) and 3 (1-7) for confirmed and suspected COVID-19 cases, respectively. The highest death rate was observed among ages>65 (63.4% of confirmed cases, 62.3% of suspected cases) and intensive care unit (ICU)/critical care unit (CCU) patients (62.7% of confirmed cases, 52.2% of suspected cases). Total case fatality rate (CFR) was 10.05% (13.52% and 6.37% among confirmed and suspected cases, respectively). The highest total CFR was observed in patients with age>65 years (25.32%), underlying comorbidities (25.55%), and ICU/CCU patients (41.7%). The highest CFR was reported for patients who had diabetes and cardiovascular diseases (38.46%) as underlying non-communicable diseases (NCDs), and patients with cancer (35.79%).

**Conclusion:**

This study showed a high CFR among suspected and confirmed COVID-19 cases, and highlighted the main associated risk factors including age, sex, underlying NCDs, and ICU/CCU admission affecting survival of COVID-19 patients.

## Introduction

The COVID-19 pandemic is currently known as an ongoing pandemic of one the most fatal types of infectious diseases [1, 2]. The disease was first reported in Wuhan, Hubei, China in December 2019. After SARS coronavirus (SARS-CoV) in 2002 and Middle East respiratory syndrome coronavirus (MERS-CoV) in 2012, the COVID-19 outbreak is recognized as the third outbreak of coronavirus since two decades ago [3]. The pandemic was caused by severe acute respiratory syndrome coron3virus 2 (SARS-CoV-2) [4] affecting more than 5.5 million people all over the world up to now [5/26/2020]. More than 340,000 of the population infected by COVID-19 faced death in all countries [5]. Europe and the Unites States of America reported the highest incidence and mortality rates of COVID-19 among all countries with almost 2 million cases and 168,917 deaths in Europe and over 1.7 million patients with COVID-19 in the US and around 100,000 deaths in the US [5, 6]. The first confirmed COVID-19 case in Iran was reported on February 20, 2020, in Qom province [7] and until Aug 2 2020, a total of 306,752 confirmed COVID-19 cases were reported and 16,982 patients had died due to COVID-19 in Iran [8]. According to the most recently updated global statistics, Iran currently ranks 10th in the world and second in the middle east in terms of the number of deaths due to COVID-19 [5].

Recent studies have found that several risk factors could be associated with an increased risk of death among COVID-19 cases, which should be taken into account when it comes to control and management of the pandemic [6, 9, 10]. Among these risk factors, pre-existing non-communicable comorbidities such as cancers, diabetes, hypertension, cardiovascular diseases, chronic kidney diseases, chronic pulmonary diseases, and other chronic diseases could be regarded to be the most important risk factors of death in COVID-19 patients [9, 11, 12]. Therefore, assessing these characteristics could help decision-makers, clinicians, and scholars to decrease the burden of COVID-19 disease.

At the time of an outbreak of infectious diseases, such as COVID-19, case fatality rate (CFR) can help epidemiologists and health care experts to identify abnormalities present in a population and help researchers estimate mortality rates due to the disease early on [13-15]. However, performing this quantification could be hampered by issues such as extent of testing, as lack of an accurate estimation of CFR in the early stages of a pandemic may result in underestimation of mortality rates [14]. Nevertheless, employing this indicator could improve the estimation of fatality rate and monitoring its general, and help estimate the spread of the infection in a society. Based on a recent study conducted in Iran, CRF had a downward trend until March 26, 2020 [16], but changes in the number of cases since March made substantial changes to this indicator, which should be taken into consideration.

Exploring the epidemiological features of COVID-19 and assessing underlying comorbidities among affected patients could help public health officials, decision makers and clinicians to take initiative in reducing the burden of this infectious disease and consequently control the epidemic. Although several studies have been recently performed to report certain epidemiological characteristics of the disease in the US, Europe and mainly china [17-22], few studies have assessed these important factors in Iranian populations [23-26]. This study aimed to evaluate some of the epidemiological and clinical characteristics as well as associated Factors of Mortality in a sample of the Iranian population, by studying confirmed and suspected cases of COVID-19 among all those who were hospitalized in public hospitals and medical centers affiliated to Shahid Beheshti University of Medical Sciences (SBMU), Tehran, Iran.

## Methods

**2-1. Study design and participants**

This cross-sectional study was performed in 19 hospitals in Tehran, Iran, which were designated as COVID-19 medical centers, including Akhtar, Ayatollah Ashrafi Esfahani, Imam Hossein, 15 Khordad, Zaeem, Shahid Sattari, Sevom-e-Shaban, Shohadaye Pakdasht, Shohadaye Tajrish, Shohadaye Gomnam, Ayatollah Taleghani, Torfeh, Imam Khomeini (Firoozkooh), Loghman Hakim, Shahid Modarres, Masih Daneshvari, Shahid Mofateh(Varamin), Mofid Children's, and Mahdieh. All the mentioned hospitals are affiliated to SBMU and are responsible for 52% of health care services in Tehran province. All patients with positive polymerase chain reaction (PCR) test results admitted and hospitalized as confirmed COVID-19 cases between February 19 and May 12, 2020 were studied. In addition to laboratory-confirmed SARS-CoV-2 cases, patients with clinical sings of COVID-19 on chest computed tomography (CT) scan findings (presence of Ground-glass opacity pattern, consolidation, reticular pattern, or mixed pattern and honeycomb pattern) who initially had negative PCR test results were diagnosed as highly suspected COVID-19 patients and hospitalized. The study was approved by Research Ethics Committee of SBMU, Tehran, Iran (IR.SBMU.RETECH.REC.1399.486). 

**2-2. Definitions**

Survival time was defined as the time interval between hospital admission and discharge (survived or deceased). Survivors were defined as patients who were alive at the time of discharge from the hospital. CFR, also called case fatality rate or case fatality ratio, in epidemiology, is the proportion of people who die from a specified disease among all individuals diagnosed with the disease over a certain period of time. In this study, case fatality rate was calculated by dividing the number of deaths from COVID-19 disease over a defined period of time by the number of individuals diagnosed with the disease during that time (PCR &CT). The deceased cases were patients who died due to COVID-19 during the study period [27].

**2-3. Data collection and preparation**

The epidemiological and demographic data were extracted from Hospital Intelligent Management (HIM) system. HIM system is a comprehensive and integrated decision-making system. In this system, hospital performance information is integrated from various databases, and then it is provided to managers in the form of performance reports and specialized dashboards.

The information extracted, from both the laboratory-confirmed SARS-CoV-2 and suspected cases, included underlying comorbidities, laboratory and radiological findings, treatment and death outcome during hospitalization and recovery status (complete or partial), which are displayed in the HIM system. 

Data quality control was performed in several steps. Duplicate records were identified based on patients’ national identification code. For patients without a national code in the HIM system, first name and surname, admission code, patient’s address, age, and other registered information were checked. Patients with the same information in all mentioned characteristics were identified as a duplicate record and removed from the dataset. If the patient was admitted at different times, the first date of referral was considered as the first time of infection. Patients who had records as both confirmed and suspicious case, their confirmed status was considered as the final diagnosis. Any missing or unknown records in the HIM system were collected and clarified through checking the Hospital medical records units, and asking from patients and their families. 

**2-4. Statistical analysis**

Frequency (percentage) was reported for all categorical variables in survivors, the deceased, and total cases. Continuous variables with symmetric and skewed distribution were described as mean (standard deviation (SD)) and median (first quantile-third quantile). Chi-square, independent test, and fisher’s exact test were used to determine if there is a significant relationship between categorical variables and status (deceased or survivor). Independent t-test and Mann-Whitney U test were used to compare the mean and median of continuous variables between survivors and deceased cases, respectively. CFRs were calculated for categorical variables based on status. The unadjusted and adjusted Cox proportional-hazard models were applied to determine the impact of variables on the survival time of cases and results were reported using hazard ratio (HR) and 95% confidence interval (CI). Statistical analyses were done using IBM SPSS version 26 and p-value less than 0.05 was considered as statistically significant. 

## Results

***Baseline characteristics of studied cases***

**Figure 1:**
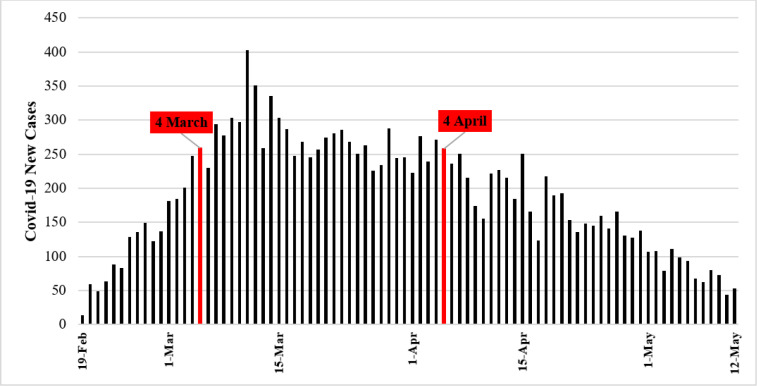
The distribution of daily new admitted cases of COVID-19 from February to May 2020 in hospitals affiliated to Shahid Beheshti University of Medical Sciences, Tehran, Iran

From February 19, 2020 to May 12, 2020, 8252 confirmed COVID-19 cases and 7783 individuals diagnosed as suspected cases of COVID-19 had referred to hospitals affiliated to SBMU, Iran, Tehran. Results showed that 8246 (99.93%) of the confirmed cases and 7770 (99.83%) of the suspected cases had been hospitalized and the rest had received outpatient health care services. Figure 1 shows the distribution of daily new cases of COVID-19 during the study period. 

Evaluation of all inpatients revealed that 1116 (13.52%) of confirmed and 506 (6.37%) of suspected COVID-19 cases, had died during 5 (IQR=2-9) and 3 (IQR=1-7) days of hospitalization, respectively. The age of patients significantly correlated with survival status in both confirmed and suspected cases (p < 0.001). The mean (SD) age of confirmed COVID-19 patients who died was 67.54 (15.81) years, while this value was 49.17 (17.43) years for recovered cases. In addition, suspected cases who died due to COVID-19 had the mean age of 66.65 (19.52) while mean age of the suspected survivors was 47.65 (18.99) years. Most of the new cases (57.2 % of confirmed cases and 56.9% of suspected cases) and patients who died from COVID-19 (63.4% of confirmed cases and 59.5% of suspected cases) were male in our study (Table 1). 

***Case fatality rate***

The overall CFR was 10.05%, while the rate was 13.52% and 6.37% for confirmed and suspected cases of COVID-19, respectively. Seniors had higher CFR compared to other age categories with 32.49% for confirmed and 16.82% for suspected cases of COVID-19. CFR was reported to be 34.19% among confirmed cases of COVID-19 with underlying comorbidities, which was about 3 times higher than those without any comorbidity. The highest CFRs reported among confirmed COVID-19 cases belonged to patients with diabetes and cardiovascular diseases (66.67%), unspecified incurable disease (54.55), and cancers (46.43%). However, CFRs among suspected cases of COVID-19 with unspecified incurable disease (71.43%), diabetes and hypertension (26.83%) and cancers (20.51%) were higher than those in other groups of underlying morbidities. Patients with confirmed COVID-19 admitted to ICU/CCU had a CFR of 59.68%, whereas this rate was 22.29% among suspected patients in the ICU/CCU (Table 2). 

***Associated factors of Mortality***

**Figure 2:**
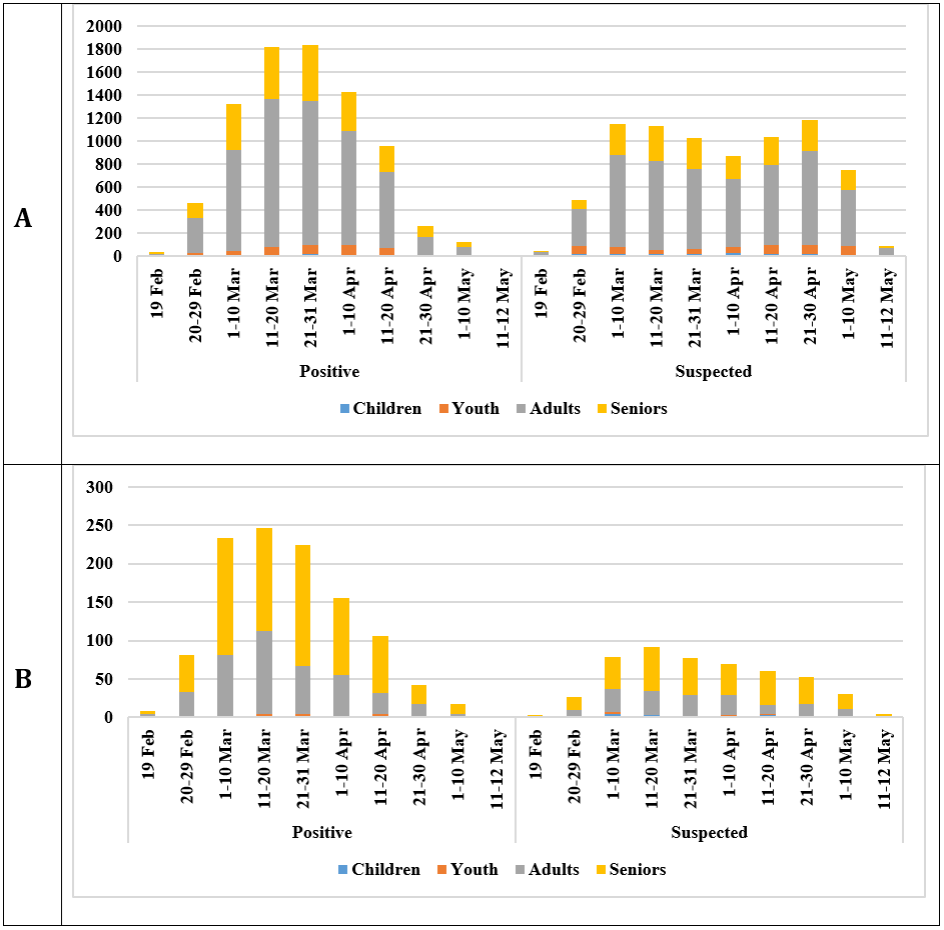
The distribution of A) number of new cases, and B) number of deaths based on age group and time periods among COVID-19 cases admitted to hospitals affiliated to Shahid Beheshti University of Medical Sciences, Tehran, Iran, from February to May 2020

Survival status was associated with age group (p < 0.001), sex (p < 0.001; only for confirmed COVID-19 cases), underlying comorbidities (p < 0.001), type of hospital admission (p < 0.001), and hospital unit (p< 0.001). 22.7% of confirmed cases and 14.7% of suspected cases who died from COVID-19 had underlying comorbidities. Overall, the most frequent comorbidities were diabetes mellitus (2.5% among confirmed cases; 1.5% among suspected cases) and cardiovascular disease (2.4% of confirmed cases; 1.5% of suspected cases), both of which had a high prevalence among deceased patients. The highest percentage of death due to COVID-19 was found in ICU/CCU patients with 62.7% of confirmed cases and 52.2% of suspected cases (Table 1). Figure 2 indicates that the highest number of new COVID-19 cases were found in the group of adults (25-64 years old) since the beginning of the outbreak, while the highest frequency of death was reported in seniors (>64 years old). The results of multiple Cox proportional hazard regression analysis revealed that being male (adjusted HR: 1.19; 95%CI: 1.05-1.35), age≥65 (adjusted HR: 2.18; 95%CI: 1.93-2.48), and admission to ICU/CCU wards (adjusted HR: 3.93; 95%CI: 3.46-4.57) were significant risk factors associated with survival time of confirmed cases who died due to COVID-19 during hospitalization. The significant risk factors associated with survival time of deceased suspected cases of COVID-19 were age≥65 (adjusted HR: 2.38; 95%CI: 1.97-2.87), underlying comorbidities (adjusted HR: 0.58; 95%CI: 0.75-0.75) and admission to ICU/CCU wards (adjusted HR: 3.52; 95% CI:2.94-4.22; Table 3). 

**Table 1:** Epidemiological, demographic and clinical characteristics of inpatient confirmed and suspected COVID-19

**Variables**	**Confirmed cases**			**Suspected cases**		
	**Survivors n=7136**	**Deceased n=1116**	**P**	**Survivors n=7287**	**Deceased n=496**	**P**
**Age, years**						
Mean (SD)	49.17 (17.43)	67.54 (15.81)	<0.001	47.66 (18.99)	66.65 (19.52)	<0.001
Median (range)	48.00 (0-99)	70.00 (3-97)	<0.001	46.00 (0-105)	71.00 (0-102)	<0.001
Children (0-14)	73/7136 (1.0)	5/1116 (0.4)	<0.001	170/7287 (2.3)	14/496 (2.8)	<0.001
Youth (15-24)	356/7136 (5.0)	10/1116 (0.9)		488/7287 (6.7)	7/496 (1.4)	
Adults (25-64)	5238/7136 (73.4)	394/1116 (35.3)		5101/7287 70.0)	166/496 (33.5)	
Seniors (>65)	1469/7136 (20.6)	707/1116 (63.4)		1528/7287 21.0)	309/496 (62.3)	
**Sex**			<0.001			0.228
Male	4010/7136 (56.2)	708/1116 (63.4)		4132/7287(56.7)	295/496 (59.5)	
Female	3126/7136 (43.8)	408/1116 (36.6)		3155/7287 (43.3)	201/496 (40.5)	
**Underlying comorbidity**			<0.001			<0.001
Yes	487/7136 (6.8)	253/1116 (22.7)		463/7287 (6.4)	73/496 (14.7)	
No	6649/7136 (93.2)	863/1116 (77.3)		6824/7287 (93.6)	423/496 (85.3)	
**Underlying comorbidity (Type)**			<0.001			<0.001
No Disease history	6649/7136 (93.2)	863/1116 (77.3)		6824/7287 (93.6)	423/496 (85.3)	
Diabetes Mellitus (DM)	155/7136 (2.2)	53/1116 (4.7)		104/7287 (1.4)	13/496 (2.6)	
Cardiovascular Disease	130/7136 (1.8)	66/1116 (5.9)		99/7287 (1.4)	17/496 (3.4)	
Pulmonary Disease	44/7136 (0.6)	31/1116 (2.8)		73/7287 (1.0)	4/496 (0.8)	
Hypertension (HTN)	47/7136 (0.7)	25/1116 (2.2)		61/7287 (0.8)	9/496 (1.8)	
Cancer	30/7136 (0.4)	26/1116 (2.3)		31/7287 (0.4)	8/496 (1.6)	
DM and HTN	32/7136 (0.4)	18/1116 (1.6)		30/7287 (0.4)	11/496 (2.2)	
Neurological Disease	16/7136 (0.2)	12/1116 (1.1)		15/7287 (0.2)	4/496 (0.8)	
Kidney Disease	12/7136 (0.2)	1/1116 (0.1)		28/7287 (0.4)	1/496 (0.2)	
Unspecified Incurable	5/7136 (0.1)	6/1116 (0.5)		2/7287 (0.0)	5/496 (1.0)	
DM and Cardiovascular	2/7136 (0.0)	4/1116 (0.4)		6/7287 (0.1)	1/496 (0.2)	
Anemia	3/7136 (0.0)	2/1116 (0.2)		5/7287 (0.1)	0/496 (0.0)	
Others*	11/7136 (0.2)	9/1116 (0.8)		9/7287 (0.1)	0/496 (0.0)	
**Pregnancy**			0.349			1.000
Yes	7/7136 (0.1)	2/1116 (0.2)		6/7287 (0.1)	0/496 (0.0)	
No	7129/7136 (99.9)	1114/1116(99.8)		7281/7287 (99.9)	496/496(100.0)	
**Type of Hospital Admission**			<0.001			<0.001
Hospitalization	3967/7136 (55.6)	1043/1116 (93.5)		2957/7287 (40.6)	434/496 (87.5)	
Temporary	3163/7136 (44.3)	73/1116 (6.5)		4317/7287 (59.2)	62/496 (12.5)	
Outpatient	6/7136 (0.1)	0/1116 (0.0)		13/7287 (0.2)	0/496 (0.0)	
**Wards**			<0.001			<0.001
ICU and CCU	473/7136 (6.6)	700/1116 (62.7)		903/7287 (12.4)	259/496 (52.2)	
Others	6663/7136 (93.4)	416/1116 (37.3)		6384/7287 (87.6)	237/496 (47.8)	
**Duration of hospitalization (days)**						
Median (Q1-Q3)	1 (0-5)	5 (2-9)	<0.001	0 (0-2)	3 (1-7)	<0.001

Data are expressed as mean (standard deviation), median (Q1-Q3) and frequency (percentage) for symmetric numeric variables, asymmetric numeric variables and categorical variables, respectively. P-values are used for comparing mean or median of numeric variables between survivors and deceased patients using independent t-test and Mann-Whitney U test. The p-values for categorical variable are for assessing the independence assumption between patients’ status (survivors versus deceased) and categorical variables. * Others group in underlying comorbidities included AIDS, autoimmune disorders, gastrointestinal disease, war injuries, tuberculosis, thyroid disease, and hepatitis.

**Table 2:** Case fatality rate of patients hospitalized due to COVID-19

**Variable**	**All cases n=16035**	**Confirmed n=7287**	**Suspected n=496**
**Age, years**			
Children (0-14)	7.25	6.41	7.61
Youth (15-24)	1.97	2.73	1.41
Adults (25-64)	5.14	7.00	3.15
Seniors (>65)	25.32	32.49	16.82
**Sex**			
Male	10.97	15.01	6.66
Female	8.84	11.54	5.99
**Underlying comorbidity**			
Yes	25.55	34.19	13.62
No	8.71	11.49	5.84
**Underlying comorbidity (Type)**			
No Disease history	8.71	11.49	5.84
Diabetes Mellitus (DM)	20.31	25.48	11.11
Cardiovascular Disease	26.60	33.67	14.66
Pulmonary Disease	23.03	41.33	5.19
Hypertension	23.94	34.72	12.86
Cancer	35.79	46.43	20.51
DM and Hypertension	31.87	36.00	26.83
Neurologic Disease	34.04	42.86	21.05
Kidney Disease	4.76	7.69	3.45
Unspecified Incurable	61.11	54.55	71.43
DM and Cardiovascular	38.46	66.67	14.29
Anemia	20.00	40.00	0.00
Others*	31.03	45.00	0.00
**Pregnancy**			
Yes	13.33	22.22	0.00
No	10.05	13.51	6.38
**Type of Hospital Admission**			
Hospitalization	17.58	20.82	12.80
Temporary	1.77	2.26	1.42
Outpatient	0.00	0.00	0.00
**Hospital Unit**			
CCU/ICU	41.07	59.68	22.29
Others	4.77	5.88	3.58
**Total**			
%	10.05	13.52	6.37

* Others group in underlying comorbidities included AIDS, autoimmune disorders, gastrointestinal disease, war injuries, tuberculosis, thyroid disease and hepatitis.

**Table 3:** Unadjusted and adjusted cox proportional hazards regression model for death during hospitalization

**Group**	**Variable**	**Unadjusted Hazard**			**Adjusted Hazard**		
		**HR**	**95% CI**	**P value**	**HR**	**95% CI**	**P value**
**Confirmed Case**	Sex male vs. female	1.23	1.09-1.39	0.001	1.19	1.05-1.35	0.005
	Age, ≥65 years vs. <65 years	2.78	2.46-3.14	<0.001	2.18	1.93-2.48	<0.001
	Underlying, yes vs. no	1.20	1.04-1.39	0.003	0.90	0.78-1.03	0.895
	Hospital wards, ICU/CCU vs. others	4.57	4.04-5.17	<0.001	3.93	3.46-4.57	<0.001
	Pregnancy, yes vs. no	0.57	0.17-2.73	0.381	---	---	---
**Suspected Cases**	Sex, male vs. female	1.15	0.96-1.38	0.120	---	---	---
	Age, ≥65 years vs. <65 years	2.69	2.23-3.24	<0.001	2.38	1.97-2.87	<0.001
	Underlying, yes vs. no	0.70	0.54-0.90	0.006	0.58	0.45-0.75	<0.001
	Hospital wards, ICU/CCU vs. others	3.90	3.26-4.67	<0.001	3.52	2.94-4.22	<0.001
	Pregnancy, yes vs. no	---	---	---	---	---	---
**All patients**	Sex, male vs. female	1.22	1.10-1.35	<0.001	1.22	1.11-1.35	<0.001
	Age, ≥65 years vs. <65 years	2.71	2.45-3.01	<0.001	2.21	1.99-2.45	<0.001
	Underlying, yes vs. no	1.03	0.91-1.16	0.682	---	---	---
	Hospital wards, ICU/CCU vs. others	4.36	3.95-4.84	<0.001	3.75	3.37-4.16	<0.001
	Pregnancy, yes vs. no	0.60	0.15-2.40	0.468	---	---	---
	COVID-19 Status, Confirmed vs suspected	1.21	1.09-1.35	<0.001	1.13	1.02-1.26	0.022

CI: confidence interval; HR: hazard ratio.^ * ^All significant variables in the unadjusted model were included in the adjusted model. Other variables were removed from the adjusted model.

## Discussion

To make decisions on interventions and regulations for controlling COVID-19 pandemic, an accurate estimation of epidemiologic rates is required, especially infection and death rates and associated comorbidities. Here, by integrating data from nineteen hospitals, we found that almost half of the patients were considered as suspected COVID-19 cases and others were confirmed cases who were positive for SARS-CoV2 in PCR. In addition, our study showed that the peak of COVID-19 outbreak occurred in a one-month interval between March 4th and April 4th, 2020. In addition, age, sex, underlying comorbidity, hospitalization in the ICU/CCU, and duration of hospitalization were shown to be significant associated risk factors. Survival analysis was performed to evaluate the effect of risk factors on death outcome during hospitalization. 

In both confirmed and suspected groups, risk factors including old age, underlying disease, ICU/CCU admission, and duration of hospitalization were significantly associated with patient status (Deceased vs. survivors) while sex was associated only with confirmed COVID-19. In recent studies, age>60 years has been identified as a major risk factor [28-31]. Moreover, studies have shown that risk of dying from COVID-19 in men is higher than women [28, 31-33]. The relationship between having at least one underlying comorbidity and death rate was also investigated and confirmed [28, 31-35]. The national epidemiologic reports indicate an association between death rate and both age>60 years and having at least one underlying comorbidity [36, 37]. Li et al. also showed that a longer duration of hospitalization was associated with a higher risk of death [28]. 

Our findings showed that although about 70% of suspected and confirmed COVID-19 cases were in the age of 25-64 years, about 60% of deaths occurred in patients over 65 years old. In addition, approximately 50% of all deaths occurred in ICU/CCUs patients. According to our results, almost half of the underlying non-communicable diseases (NCDs) and related deaths were related to diabetes mellitus and cardiovascular diseases. In a study conducted by Hongdou et al. on 5139 infected patients, although 80% of patients were 25-64 years old, approximately 60% of deaths occurred in those aged more than 64 years [38]. Similar results were reported in Zhonghua et al. study, which included 72314 COVID-19 patients [39]. Few studies with large sample size have reported death rates due to COVID-19 in ICU patients. In a study carried out in the United States evaluating the patients records in 12 hospitals, 50% of deaths had occurred in the ICUs [40] . In a systematic review, the average CFR reported for patients admitted to ICU was 25.7% during hospitalization [41]. Similar to the findings of our study, cancer, diabetes, hypertension, and cardiovascular diseases were the most common underlying comorbidities among COVID-19 cases in various studies [10, 19-21]. In Guan et al.'s study, about 70% of patients had diabetes and more than 50% of all deaths were reported among diabetic patients [34].

In our study, CFR was 13.52% among confirmed COVID-19 cases, which is almost twice as high as in suspected cases. According to a national study, the cumulative risk for in-hospital mortality in 30 days was 24.4% [42]. The CFR in confirmed cases of our study was about 10% higher than that reported by the study of Chinese Center for Disease Control and Prevention with 2.3% (36). But according to data from the New York City Department of Health and Mental Hygiene in May 12, 2020, the CFR in confirmed cases in New York, the most populous city in the United States, was 6.8% [43]. One reason for this difference in results might be that CFR has been calculated among hospitalized patients in our study, while other studies considered all patients, either hospitalized or not.

CFR indicates the lethality of the disease and only includes the ratio of deaths to patients, and is especially used in acute infectious diseases. CFR may be different in different epidemics because there are changes in the pathogen, host, and environment. CFR is closely related to the virulence of the pathogen. Given the mutations and changes in the coronavirus and the higher CFR in patients with underlying NCDs, we highly recommend preventing precautions and screening people with underlying diseases with a focus on NCDs.

We also used unadjusted and adjusted cox proportional hazard for exploring factors associated to COVID-19 deaths. Our findings showed that male sex, age 65 years or older, and ICU/CCU admission were risk factors of the survival of COVID-19 patients. In addition, confirmed COVID-19 patients had a significantly higher risk of death compared to suspected cases. Few studies have reported the risk factors for survival of COVID-19 patients. In a study by Li et al., male sex and age group of 65 years or older had a significant negative effect on the survival of COVID-19 [25]. Wang et al. studied 538 confirmed cases of COVID-19 and showed that older age was associated with an increase in hospitalization [44]. Unfortunately, no study was performed to compare the survival rate between confirmed and suspected COVID-19 cases. There are no clear reasons for the higher risk of death among confirmed COVID-19 cases compared to suspected ones. Cases who were confirmed could have shown more severe symptoms of the disease and were, therefore, more likely to experience death.

According to the findings, we highly recommend screening people with underlying NCDs in the second phase of the epidemics in order to reduce death rates through early detection and provision of proper care. We also suggest adopting a response plan to manage underlying NCDs in the context of COVID-19 epidemic. Given the high CFR found in this study and the unknown behavior of coronavirus, there is room for more discussions and more data are required. 

## Limitations

This study was the largest multicenter study ever conducted in Tehran province, Iran, on patients with COVID-19, evaluating the two groups of suspected and confirmed cases of COVID-19. However, our data was limited to the public hospitals affiliated to SBMU. 

## Conclusion

This study showed the high CFR for confirmed COVID-19 cases and the difference in CFRs of suspected and confirmed cases. It also highlighted age, sex, underlying NCDs, ICU/CCU admission, and confirmed/suspected status as associated risk factors of mortality among COVID-19 patients. 
